# The Role of Cholecystokinin in Peripheral Taste Signaling in Mice

**DOI:** 10.3389/fphys.2017.00866

**Published:** 2017-10-31

**Authors:** Ryusuke Yoshida, Misa Shin, Keiko Yasumatsu, Shingo Takai, Mayuko Inoue, Noriatsu Shigemura, Soichi Takiguchi, Seiji Nakamura, Yuzo Ninomiya

**Affiliations:** ^1^Section of Oral Neuroscience, Graduate School of Dental Sciences, Kyushu University, Fukuoka, Japan; ^2^OBT Research Center, Graduate School of Dental Sciences, Kyushu University, Fukuoka, Japan; ^3^Section of Oral and Maxillofacial Oncology, Division of Maxillofacial Diagnostic and Surgical Sciences, Faculty of Dental Science, Kyushu University, Fukuoka, Japan; ^4^Division of Sensory Physiology, Research and Development Center for Taste and Odor Sensing, Kyushu University, Fukuoka, Japan; ^5^National Kyushu Cancer Center, Institute for Clinical Research, Fukuoka, Japan; ^6^Monell Chemical Senses Center, Philadelphia, PA, United States

**Keywords:** cholecystokinin, bitter taste, neurotransmitter, gastrointestinal hormone, gustatory responses

## Abstract

Cholecystokinin (CCK) is a gut hormone released from enteroendocrine cells. CCK functions as an anorexigenic factor by acting on CCK receptors expressed on the vagal afferent nerve and hypothalamus with a synergistic interaction between leptin. In the gut, tastants such as amino acids and bitter compounds stimulate CCK release from enteroendocrine cells via activation of taste transduction pathways. CCK is also expressed in taste buds, suggesting potential roles of CCK in taste signaling in the peripheral taste organ. In the present study, we focused on the function of CCK in the initial responses to taste stimulation. CCK was coexpressed with type II taste cell markers such as Gα-gustducin, phospholipase Cβ2, and transient receptor potential channel M5. Furthermore, a small subset (~30%) of CCK-expressing taste cells expressed a sweet/umami taste receptor component, taste receptor type 1 member 3, in taste buds. Because type II taste cells are sweet, umami or bitter taste cells, the majority of CCK-expressing taste cells may be bitter taste cells. CCK-A and -B receptors were expressed in both taste cells and gustatory neurons. CCK receptor knockout mice showed reduced neural responses to bitter compounds compared with wild-type mice. Consistently, intravenous injection of CCK-Ar antagonist lorglumide selectively suppressed gustatory nerve responses to bitter compounds. Intravenous injection of CCK-8 transiently increased gustatory nerve activities in a dose-dependent manner whereas administration of CCK-8 did not affect activities of bitter-sensitive taste cells. Collectively, CCK may be a functionally important neurotransmitter or neuromodulator to activate bitter nerve fibers in peripheral taste tissues.

## Introduction

Cholecystokinin (CCK) is a gastrointestinal hormone secreted from enteroendocrine I cells, which stimulates gastric emptying in addition to the secretion of digestive enzymes and bile from the pancreas and gallbladder (Moran and Kinzig, 2004). It also plays an important role in regulating food intake. Infusions of CCK reduce food intake in humans and animals (Gibbs et al., 1973; Kissileff et al., 1981). In addition, CCK is abundantly expressed in the brain and functions as a neurotransmitter (Innis and Snyder, 1980). There are two types of CCK receptors, CCK-Ar and CCK-Br, both of which are G protein-coupled receptors encoded by different genes (Wank, 1995). Among them, CCK-Ar is primarily responsible for controlling food intake. A specific CCK-Ar agonist potently inhibits food intake (Asin et al., 1992), whereas a specific CCK-Ar antagonist blocks the ability of CCK to inhibit food intake (Moran et al., 1992). CCK directly activates vagal afferent fibers via activation of CCK-Ar (Schwartz et al., 1994). These signals are relayed to the hypothalamus to induce inhibition of food intake. CCK-Ar is also expressed in the hypothalamus, and activation of hypothalamic CCK-Ar leads to suppression of food intake in mice (Hirosue et al., 1993).

Various nutrients stimulate CCK release in the gut. Amino acids are known to increase plasma CCK levels and reduce food intake in humans and animals (Meyer et al., 1976; Anika et al., 1977; Owyang et al., 1986; Koop and Buchan, 1992; Liddle, 1994). In the mouse intestine, CCK-positive cells possess amino acid (umami) taste receptor, taste receptor type 1, member 1 and 3 (T1R1 and T1R3; Daly et al., 2013). CCK release from the murine intestine and a murine enteroendocrine cell line, STC-1 is significantly suppressed by a rodent T1R3 inhibitor gurmarin and enhanced by inosine monophosphate. Thus, umami taste receptor T1R1/T1R3 may function as a luminal sensor for amino acids-induced CCK release. CCK is also released from the gut in response to bitter compounds. Activation of bitter taste receptors, taste receptor type 2 (T2R) families in the intestine leads to secretion of CCK that is proposed to help limit the absorption of dietary-derived bitter-tasting toxins (Jeon et al., 2008, 2011). These data demonstrate the involvement of taste signaling molecules in CCK release from the gut.

In the taste organ, CCK is expressed in a subset of taste cells (Herness et al., 2002). Taste cells expressing CCK also possess its receptor, CCK-Ar (Herness and Zhao, 2009). Exogenous application of CCK inhibits the outward K^+^ current and induces Ca^2+^ responses in isolated rat taste bud cells (Herness et al., 2002). These findings suggest that CCK may function in an autocrine manner in taste buds. Many CCK-sensitive taste cells show Ca^2+^ responses to bitter taste stimuli such as quinine and caffeine (Lu et al., 2003). Moreover, many CCK-expressing taste cells express Gα-gustducin (Herness et al., 2005) that is involved in signal transduction for bitter, sweet, and umami tastes. These data indicate that some taste receptor cells possess CCK and possibly release CCK in response to taste stimuli. However, the role of CCK expressed in taste receptor cells is currently unknown.

To study the role of CCK in the peripheral taste tissue, we examined the expression of CCK and CCK receptors in taste buds and geniculate ganglions (GGs) that contain cell bodies of gustatory nerve fibers innervating the anterior tongue. We also investigated gustatory nerve responses to various tastants in mice genetically lacking CCK-Ar and/or CCK-Br and the effect of a CCK-Ar antagonist on gustatory nerve responses to various taste stimuli. In addition, we examined whether gustatory nerves and/ or taste receptor cells could be activated by administration of CCK.

## Materials and methods

### Animals

All experimental protocols and procedures were approved by the committee for Laboratory Animal Care and Use at Kyushu University in accordance with the National Institutes of Health Guide for the Care and Use of Laboratory Animals. CCK-Ar^−/−^ mice (Takiguchi et al., 2002), CCK-Br^−/−^ mice (Nagata et al., 1996), CCK-Ar^−/−^Br^−/−^ mice (Miyasaka et al., 2002b), and gustducin-green fluorescent protein (GFP) mice (Wong et al., 1999) have been described previously. The genetic background for CCK-Ar^−/−^, CCK-Br^−/−^, CCK-Ar^−/−^Br^−/−^ and gustducin-GFP mice is C57BL/6. Wild-type (WT) controls were CCK-Ar^+/+^Br^+/+^ littermates or C57BL/6J mice. All mice were maintained in a 12/12-h light/dark cycle and had *ad libitum* access to tap water and food pellets (CE-2, CLEA Japan, Tokyo, Japan). Subjects were both male and female, 8–16 weeks of age and ranging in weight from 20 to 32 g.

### *In situ* hybridization

RNA probes were prepared for *in situ* hybridization as described previously (Shigemura et al., 2004). Primer sequences for *in situ* hybridization are shown in Table 1. RT-PCR products were purified, cloned into the pGEM T-Easy vector (Promega, Madison, WI, USA) and confirmed by sequencing and digestion with appropriate restriction enzymes. Digoxigenin (DIG)-UTP-labeled antisense and sense RNA probes were generated by *in vitro* transcription using SP6 or T7 transcription Kits (Roche, Mannheim, Germany). Frozen blocks of dissected tongue and the GG from WT mice embedded in OCT compound (Sakura Finetechnical, Tokyo, Japan) were cut into 6–7 μm-thick sections that were mounted on silane-coated glass slides. The cryosections were fixed in 4% paraformaldehyde (PFA)/PBS for 10 min at room temperature (25°C), washed with 5× standard saline citrate (SSC) for 15 min at room temperature and then prehybridized in 5× SSC/50% formamide for 2 h at room temperature. Hybridization was performed in hybridization buffer (50% formamide, 5× SSC, 5× Denhardt's solution, 500 μg/ml denatured salmon testis DNA, 250 μg/ml denatured baker's yeast tRNA, 1 mM dithiothreitol) containing 20–200 ng/ml antisense RNA probe for 18 h at 58°C. After hybridization, sections were washed twice in 5× SSC/50% formamide for 5 min each and then twice in 0.2× SSC/50% formamide for 30 min each at the same temperature used for hybridization. Subsequently, the sections were immersed in Tris-buffered saline (TBS; 50 mM Tris/HCl, pH 7.5, and 150 mM NaCl) for 5 min at room temperature, placed in blocking solution consisting of 0.5% blocking reagent (Roche) in TBS for 30 min and then incubated with anti-DIG Fab fragments conjugated with alkaline phosphatase (1:400 dilution; Roche) in the blocking solution for 60 min at room temperature. After three washes for 5 min each in TNT buffer (50 mM Tris/HCl, pH 7.5, 150 mM NaCl, and 0.05% Tween 20), sections were immersed in alkaline phosphatase buffer (100 mM Tris/HCl, pH 9.5, 100 mM NaCl, and 50 mM MgCl_2_) for 5 min. The signals were developed using nitroblue tetrazolium chloride and 5-bromo-4-chloro-3-indolylphosphate as chromogenic substrates. The reaction was stopped by rinsing the sections in Tris-EDTA buffer, followed by mounting. The signal specificities of mRNAs for each gene in taste tissues were tested using a sense probe as a negative control.

**Table 1:** Nucleotide sequences for the primers used in *in situ* hybridization.

**Gene**	**Accession No. (NCBI)**	**Forward**	**Reverse**	**Size**
CCK-Ar	NM_009827	5′-acaataaccagacggcgaac-3′	5′-cttctgcagcttgacattgc-3′	1,445
CCK-Br	NM_007627	5′-actggggaagacagtgatgg-3′	5′-taacgatggcaccaaatgag-3′	1,344
T1R3	NM_031872	5′-tgctgctatgactgcgtggac-3′	5′-aagaagcacatagcacttggg-3′	905
P_2_X_2_	NM_153400	5′-tgcacgtttgatcaggactc-3′	5′-tctgttgggaaaggaaatgg-3′	1,007
P_2_X_3_	NM_145526	5′-atgaaaaatggcttgccttg-3′	5′-ttggacaaggggagtacagg-3′	1,035

### Immunohistochemistry

Immunohistochemistry was performed as described previously (Takai et al., 2015; Yoshida et al., 2015). Dissected tongues and GGs of mice were fixed in 4% PFA/PBS for 30–45 min at 4°C. After dehydration in sucrose solutions (10% for 1 h, 20% for 1 h, and 30% for 3 h at 4°C), frozen blocks of fixed tongues and GGs were embedded in OCT compound and cut into 8 μm-thick sections that were mounted on silane-coated glass slides. After washing with TNT buffer, the sections were treated with 0.3% H_2_O_2_ in TNT buffer for 5 min, 1% blocking reagent (Roche) for 1 h at room temperature and then incubated overnight at 4°C with primary antibodies against CCK (1:200, 2145023, Chemicon, Temecula, CA, USA; 1:100, sc-21617, Santa Cruz Biotechnology, Santa Cruz, CA, USA), CCK-Ar (1:100, sc-16172, Santa Cruz Biotechnology), CCK-Br (1:100, sc-166690, Santa Cruz Biotechnology), PLCβ2 (1:200, sc-206, Santa Cruz Biotechnology), Gα-gustducin (1:200, sc-395, Santa Cruz Biotechnology), TRPM5 (1:100, ARP35242, Aviva System Biology, San Diego, CA USA), T1R3 (1:200, sc-22458; Santa Cruz Biotechnology), and/or P_2_X_2_ (APR-003, Alomone Labs, Jerusalem, Israel) in 1% blocking reagent. After washing with TNT buffer, sections were incubated for 2 h at room temperature with secondary antibodies (Peroxidase-conjugated AffiniPure donkey anti-goat IgG, 1:500, 80067, Jackson Immuno Research Laboratories, West Grove, PA, USA; Alexa Fluor 488 donkey anti-rabbit IgG, 1:300, A-21206, Life technology, Eugene, OR, USA; Alexa Fluor 488 donkey anti-mouse IgG, 1:300, A-21202, Life technology; Alexa Fluor 546 donkey anti-goat IgG, 1:300, A-11056, Life technology) in 1% blocking reagent, washed with TNT buffer and then incubated for 30 min at room temperature with tyramide-Alexa 488 or 568 substrate (Molecular Probes, Eugene, OR, USA). The immunofluorescence of labeled taste cells was observed using a laser scanning microscope (FV-1000, Olympus, Tokyo, Japan). Images were obtained using Fluoview software (Olympus). The number of positive cells was counted in GGs, and taste buds of fungiform (FP) and circumvallate papillae (CV). Image-ProPlus (Ver. 4.0, Mediacybernetics,Warrendale, PA, USA) was used to exclude artifactual signals. Cells showing a density signal greater than the mean plus two standard deviations of the density in taste cells of the negative control (primary antibodies omitted) were considered as positive.

### Solutions

Taste solutions were as follows: 100 mM NH_4_Cl, 10–1,000 mM sucrose (Suc), 10–1,000 mM NaCl, 0.1–50 mM HCl, 10–1,000 mM monopotassium glutamate (MPG), 0.1–20 mM quinine-HCl (QHCl), 1–30 mM denatonium benzoate (Den), 0.1–2 mM quinine-SO_4_ (QSO_4_), 300 mM MgSO_4_, 30 mM tetraethylammonium (TEA), 0.5 mM cyclohexamide (Cyc), 20 mM saccharin-Na (Sac), 1 mM SC45647 (SC), 500 mM glucose (Glc), 500 mM fructose (Frc), and 300 mM glycine (Gly). Tyrode solution contained 140 mM NaCl, 5 mM KCl, 1 mM CaCl_2_, 1 mM MgCl_2_, 5 mM NaHCO_3_, 10 mM HEPES, 10 mM Glucose, 10 mM sodium pyruvate; pH adjusted to 7.4 with NaOH. Chemicals were purchased from Wako Pure Chemical Industries (Osaka, Japan) or Sigma-Aldrich (St. Louis, MO, USA).

### Gustatory nerve recordings

Whole nerve responses to lingual application of tastants were recorded from the chorda tympani (CT) or the glossopharyngeal (GL) nerve as described previously (Yoshida et al., 2010; Kusuhara et al., 2013). Mice were anesthetized by an injection of sodium pentobarbital [50–60 mg/kg body weight (b.w.)] and maintained at a surgical level of anesthesia with supplemental injections of sodium pentobarbital (8–10 mg/kg b.w. approximately every hour). The anesthetic level was evaluated by testing the withdrawal reflex to a paw pinch. Under pentobarbital anesthesia, the trachea of each mouse was cannulated and then the mouse was fixed in the supine position with a head holder to allow dissection of the CT or GL nerve. The right CT nerve was dissected free from surrounding tissues after removal of the pterygoid muscle and cut at the point of its entry into the bulla. The right GL nerve was exposed by removal of the digastricus muscle and posterior horn of the hyoid bone. The GL nerve was then dissected free from underlying tissues and cut near its entrance to the posterior lacerated foramen. The entire nerve was placed on an Ag/AgCl electrode. An indifferent electrode was placed in nearby tissue. Neural activities were amplified (K-1; Iyodenshikagaku, Nagoya, Japan) and monitored on an oscilloscope and audiomonitor. Whole nerve responses were integrated with a time constant of 1.0 s and recorded on a computer using a PowerLab system (PowerLab/sp4; AD Instrument, Bella Vista, Australia). For taste stimulation of FP, the anterior one-half of the tongue was enclosed in a flow chamber of silicone rubber. For taste stimulation of CV and foliate papillae, an incision was made on each side of the animal's face from the corner of the mouth to just above the angle of the jaw, and the papillae were exposed and their trenches opened by slight tension applied through a small suture sewn in the tip of the tongue. Taste solutions were delivered to each part of the tongue by gravity flow for 30 s (CT) or 60 s (GL). The tongue was washed with distilled water (DW) for an interval of ~1 min between successive stimulation. Only responses from stable recordings (the NH_4_Cl response magnitudes at the beginning and end of each stimulation series deviated by no more than 15%) were used for data analysis. For recordings of responses to CCK-8, 0–100 μg/Kg b.w. CCK-8 (Peptide Institute, Osaka, Japan) was administrated from the femoral vein by a micro-syringe pump (ESP-32, Eicom, Kyoto, Japan) at constant speed (60 μl/min) and time (1 min). To examine the effect of a CCK-Ar antagonist, each mouse was administrated a single i.v. injection of 0–10 μg/Kg b.w. lorglumide (Sigma-Aldrich) after recording a series of control responses. CCK-8 and lorglumide were dissolved in physiological saline. At the end of experiments, animals were sacrificed by administration of an overdose of the anesthetic.

### Taste cell recording

Recording procedures were similar to those used previously (Yoshida et al., 2009, 2015). Gustducin-GFP mice were sacrificed by cervical dislocation. The anterior part of the tongue was removed and injected with 100 μl of Tyrode solution containing 0.2–0.5 mg/ml elastase (Elastin Products, Owensville, MO, USA). After incubation for 10–20 min at room temperature, the lingual epithelium was peeled and pinned out in a Sylgard coated culture dish. Individual FP taste buds with a piece of surrounding epithelium were excised from this sheet and the mucosal side was drawn into the orifice of the stimulating pipette. The residual sheet was stored at 4°C for another series of experiments. A gentle suction on the stimulating pipette was maintained to perfuse taste solutions and to hold the taste bud in place. Bath solution (Tyrode solution) was continuously flowed into the recording chamber with a peristaltic pump at ~2 ml/min. The receptor membrane was rinsed with DW at least 30 s before and after taste stimulation (15 s). Taste bud cells containing GFP were identified by confocal laser scanning microscopy (FV-300, Olympus) and were approached by a recording electrode (inner diameter ~1–3 μm, pipette resistances 1.5–3.5 MΩ). Seal resistances were typically 3–10 times the pipette resistances. Electrical signals were recorded by a high-impedance patch-clamp amplifier (Axopatch 200B; Axon Instruments, Foster City, CA, USA) interfaced to a computer (Windows XP or 7) by an analog-to-digital board (Digidata 1320A; Axon Instruments). Signals were filtered at 1 KHz, sampled at 10 KHz and stored on the hard-disk drive of a computer using pCLAMP software (Gap-Free mode; Axon Instruments) for later analysis.

### Data analysis

In the analysis of whole nerve responses, integrated whole nerve response magnitudes were measured at 5, 10, 15, 20, and 25 s (for the CT) or 5, 10, 20, 30, and 40 s (for the GL) after stimulus onset, averaged and normalized to responses to 100 mM NH_4_Cl to account for mouse-to-mouse variations in absolute responses. This relative response was used for statistical analysis. In the analysis of taste cell recordings, the number of spikes per unit time was counted throughout the recording. The mean spontaneous firing rate was calculated by averaging the number of spikes over the 10 s period (6 periods) before (control), during and after (wash out) bath application of 100 nM CCK-8. The magnitude of response to a particular stimulus was obtained by counting the total number of impulses for the first 10 s after the onset of stimulus application and subtracting the spontaneous impulse discharge. One-way and two-way ANOVA and the *post-hoc* Tukey honestly significant difference (HSD) test and paired *t-*test were used to statistically evaluate differences between genotype (WT, CCK-Ar^−/−^, CCK-Br^−/−^, and CCK-Ar^−/−^-Br^−/−^ mice), the effect of lorglumide on gustatory nerve responses and the effect of CCK-8 on activities of gustatory nerves and taste cells. Statistical analyses were performed using the statistical software package SPSS Statistics (IBM, Armonk, NY, USA).

## Results

### Expression of CCK, CCK-Ar, and CCK-Br in taste tissues and the GG

**Figure 1:**
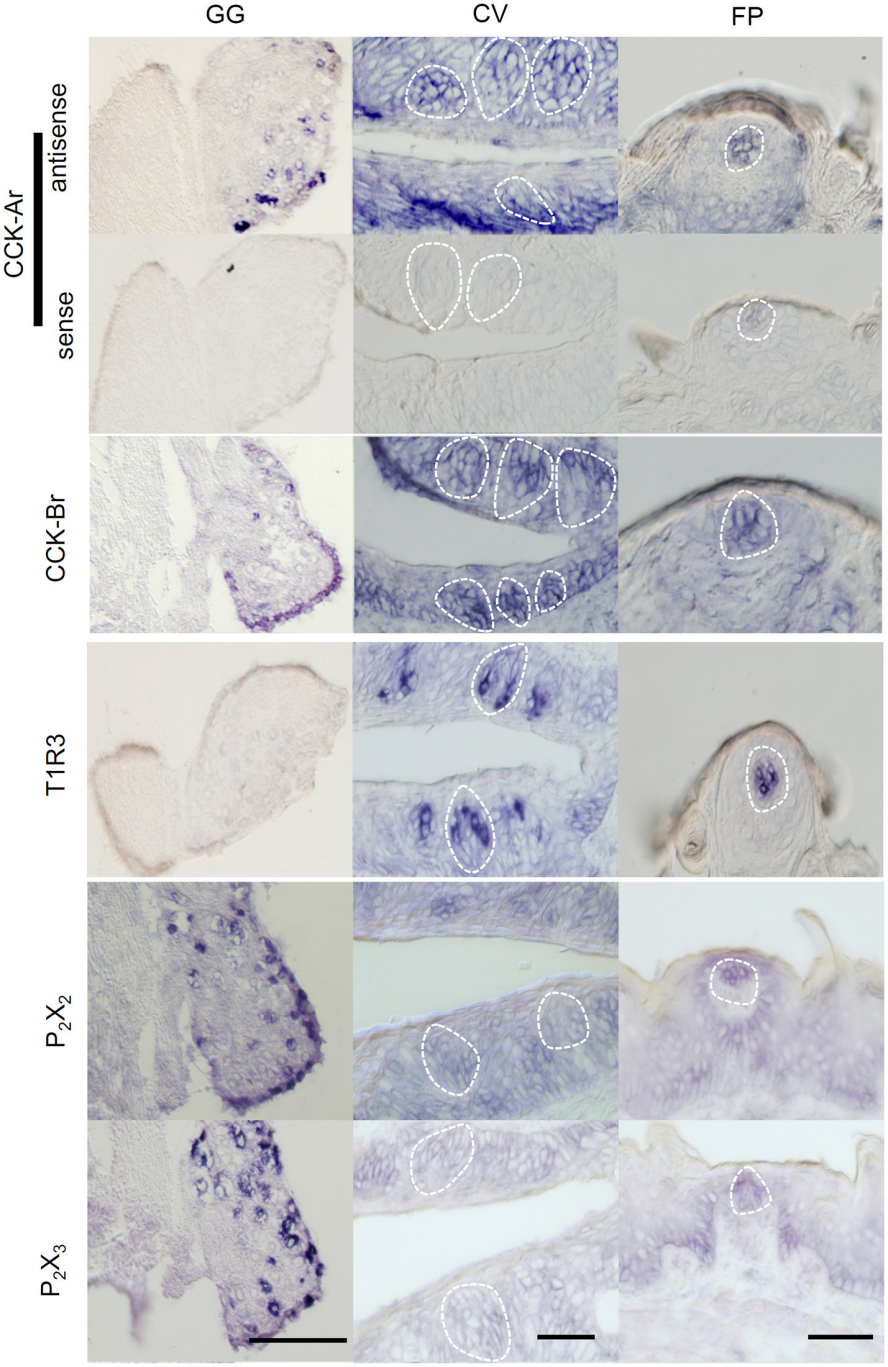
Expression of CCK-Ar and CCK-Br mRNAs in taste tissues and the geniculate ganglion (GG). Detection of mRNAs for CCK-Ar, CCK-Br, T1R3, P_2_X_2_, and P_2_X_3_ in the GG, fungiform (FP) and circumvallate papillae (CV) of C57BL/6J mice by *in situ* hybridization. The sense probe for CCK-Ar serves as a negative control. Dotted lines indicate the outline of taste buds. Scale bar, 50 μm.

Expression of CCK-Ar and CCK-Br mRNAs in taste tissues and the GG was determined by *in situ* hybridization. Both CCK-Ar and CCK-Br mRNAs were clearly detected in a subset of neurons in the GG and weakly detected in a subset of taste cells in FP and CV (Figure 1). Sweet/umami receptor component T1R3 mRNAs were detected in FP and CV, but not in the GG. Purinergic receptors P_2_X_2_ and P_2_X_3_ mRNAs were detected in the GG, but not in FP or CV. Control hybridization using a CCK-Ar sense probe was negative. These results suggest that both CCK-Ar and CCK-Br are expressed in neurons of the GG and taste cells of both the anterior and posterior tongue.

**Figure 2:**
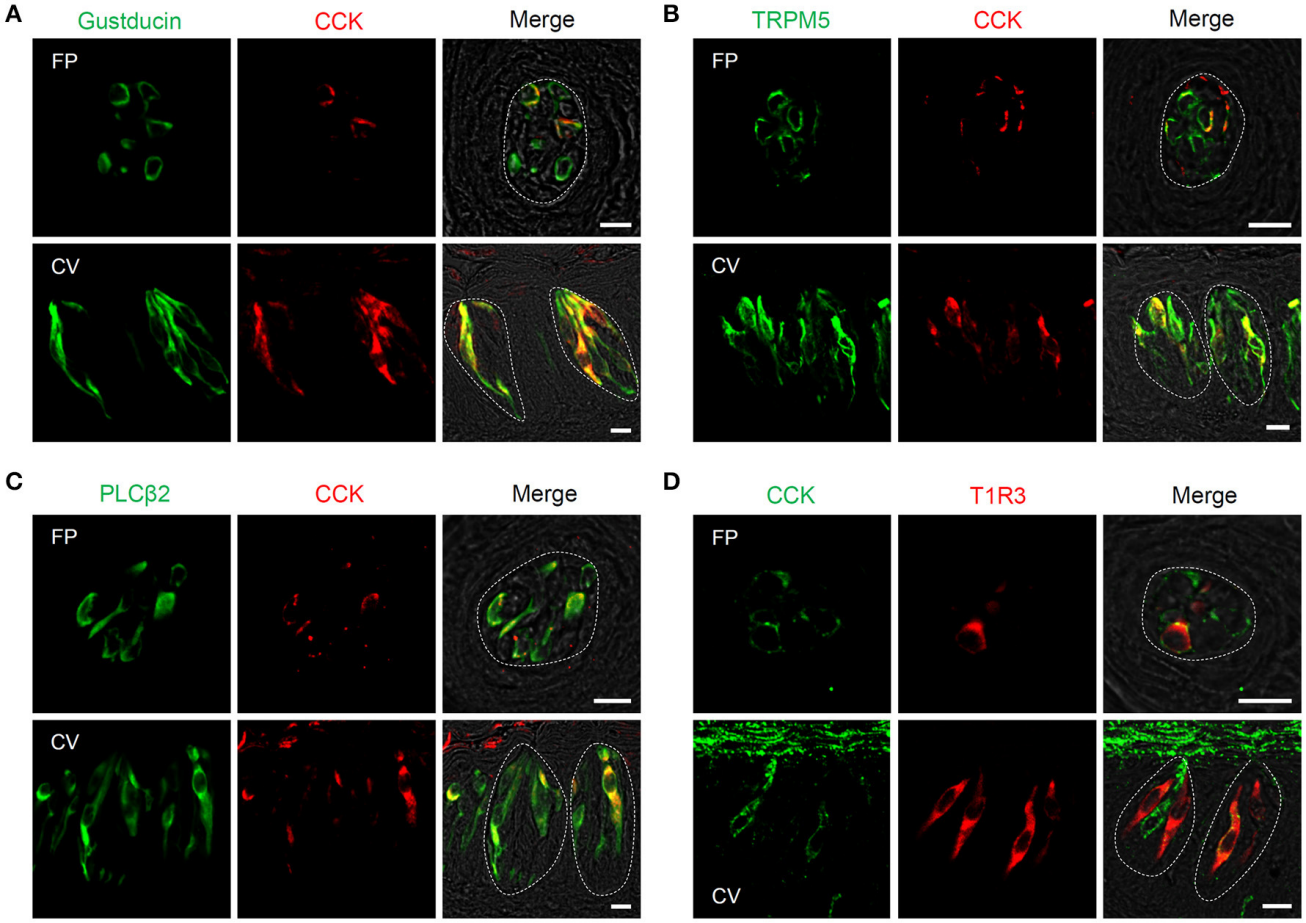
Expression of CCK and taste cell marker proteins in mouse taste bud cells. **(A–D)** Coexpression of CCK (red in **A–C**, green in **D**) and Gα-gustducin (green in **A**), TRPM5 (green in **B**), PLCβ2 (green in **C**), or T1R3 (red in **D**) in fungiform (FP) and circumvallate papillae (CV). Cells expressing both CCK and taste cell markers are shown in yellow. Dotted lines indicate the outline of taste buds. Scale bar, 10 μm.

**Figure 3:**
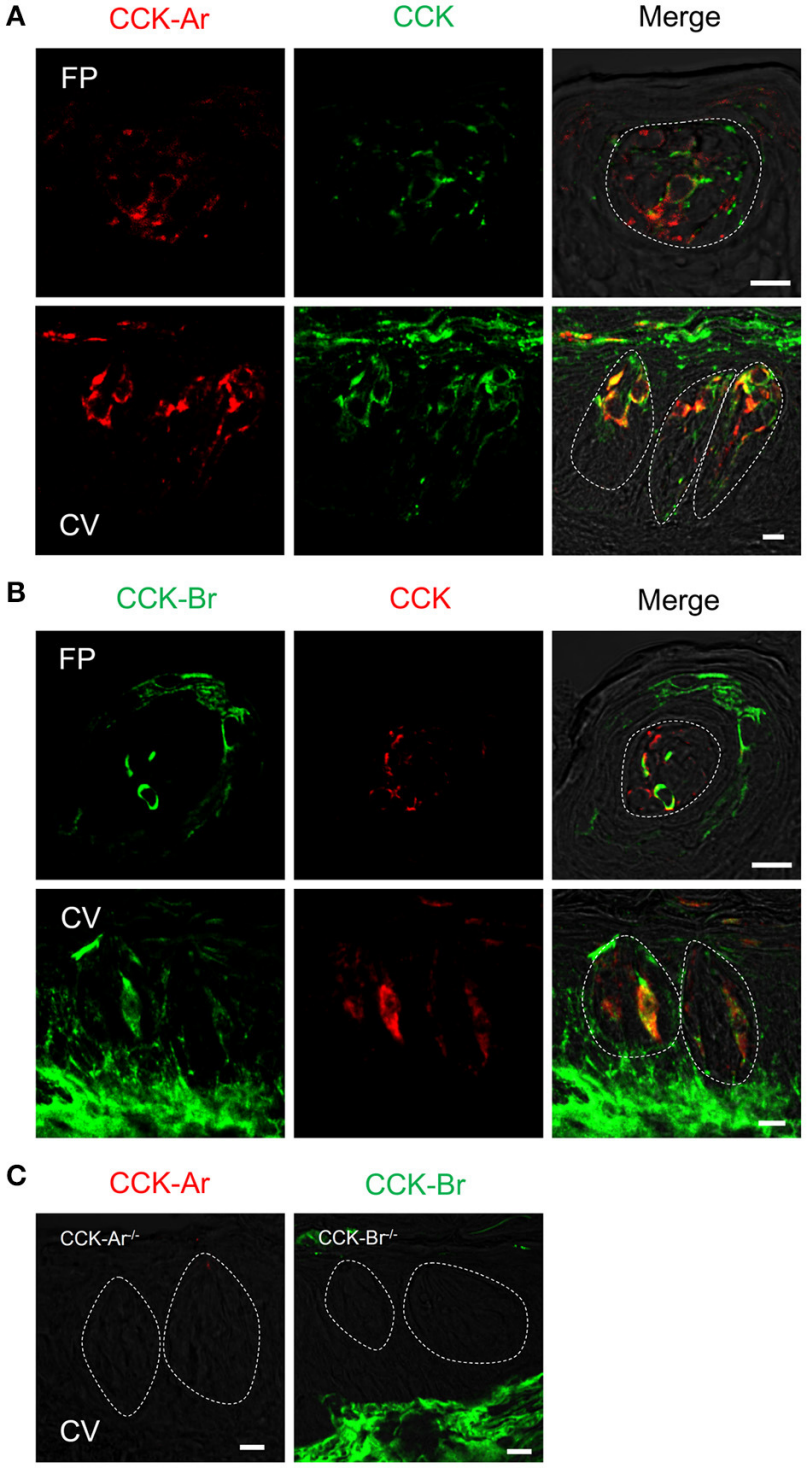
Expression of CCK, CCK-Ar, and CCK-Br proteins in mouse taste bud cells. **(A)** Coexpression of CCK (green) and CCK-Ar (red) in fungiform (FP) and circumvallate papillae (CV). **(B)** Coexpression of CCK (red) and CCK-Br (green) in FP and CV. Cells expressing both CCK and CCK receptor are shown in yellow. **(C)** Immunofluorescent images of CCK-Ar and CCK-Br in CV of CCK-Ar^−/−^ or CCK-Br^−/−^ mice, respectively. Dotted lines indicate the outline of taste buds. Scale bar, 10 μm.

**Figure 4:**
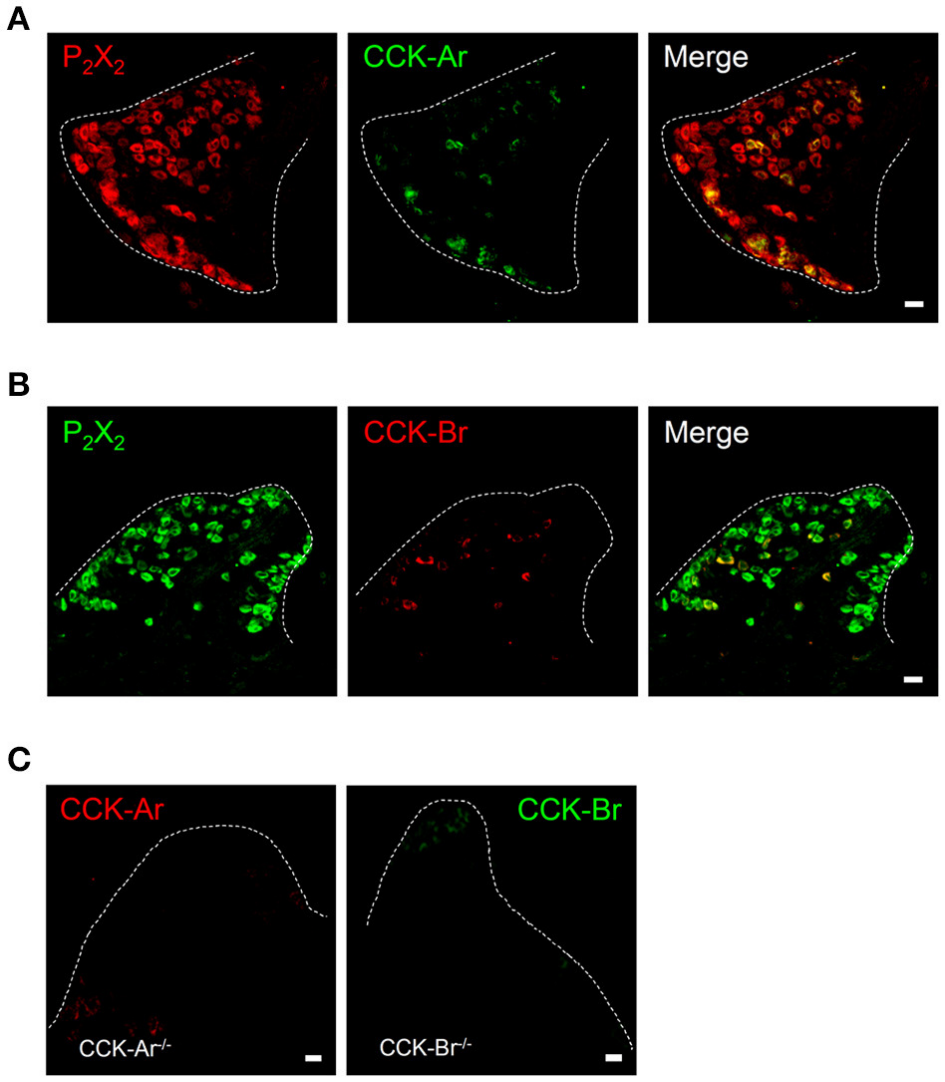
Expression of P_2_X_2_, CCK-Ar, and CCK-Br proteins in mouse geniculate ganglion (GG). **(A)** Coexpression of CCK-Ar (green) and P_2_X_2_ (red) in the GG. **(B)** Coexpression of CCK-Br (red) and P_2_X_2_ (green) in the GG. Double immunopositive cells are shown in yellow. **(C)** Immunofluorescent images of CCK-Ar and CCK-Br in the GG of CCK-Ar^−/−^ or CCK-Br^−/−^ mice. Dotted lines indicate the outline of the GG. Scale bar, 10 μm.

Next, expression of CCK, CCK-Ar, and CCK-Br proteins in FP, CV, and the GG was determined by immunohistochemistry. First, we examined coexpression of CCK and some taste cell markers in taste buds (Figure 2). Gα-gustducin (Figure 2A), TRPM5 (Figure 2B), and PLCβ2 (Figure 2C) were used as type II taste cell markers, which are expressed in sweet, umami and bitter taste cells (Iwata et al., 2014). T1R3 (Figure 2D) was used as a sweet/umami taste cell marker. Most (60–100%) CCK-immunopositive taste cells in both FP and CV were also immunopositive for type II taste cell markers Gα-gustducin, TRPM5, and PLCβ2 (Figures 2A–C, Table 2). A small subset (15–30%) of CCK-immunopositive taste cells in FP and CV were immunopositive for T1R3 (Figure 2D, Table 2). Thus, CCK-positive taste cells are likely to be type II taste cells, and the majority of them might not be sweet/umami taste cells. In addition, we examined coexpression of CCK and CCK receptors in taste buds. In both FP and CV, a subset of taste cells was immunopositive for CCK, CCK-Ar, and/or CCK-Br (Figures 3A,B, Table 3). CCK-Ar- and CCK-Br-positive taste cells were also immunopositive for CCK (Figures 3A,B, Table 3). Immunoreactivity for CCK-Ar and CCK-Br was not observed in CCK-Ar^−/−^ or CCK-Br^−/−^ mice, respectively (Figure 3C). Next we analyzed expression of CCK receptors in the GG. A subset of P_2_X_2_-positive neurons in the GG was immunopositive for CCK-Ar or CCK-Br (Figures 4A,B, Table 4). Immunoreactivity for CCK-Ar and CCK-Br was not observed in CCK-Ar^−/−^ or CCK-Br^−/−^ mice, respectively (Figure 4C). Thus, CCK receptors are expressed both in taste cells and gustatory neurons.

**Table 2:** Coexpression ratio of CCK and taste cell markers in fungiform (FP) and circumvallate papillae (CV) of mice.

**Marker**	**Fungiform**		**Circumvallate**	
CCK/Gustducin	25/39	64.1%	59/92	64.1%
CCK/TRPM5	34/54	63.0%	56/114	49.1%
CCK/PLCβ2	14/31	45.2%	87/211	41.2%
CCK/T1R3	2/7	28.6%	45/148	30.4%
Gustducin/CCK	25/27	92.6%	59/95	62.1%
TRPM5/CCK	34/47	72.3%	56/59	94.9%
PLCβ2/CCK	14/14	100%	87/95	91.6%
T1R3/CCK	2/12	16.7%	45/140	32.1%

**Table 3:** Coexpression ratio of CCK and CCK receptors in fungiform (FP) and circumvallate papillae (CV) of mice.

**Marker**	**Fungiform**		**Circumvallate**	
CCK/CCK-Ar	6/8	75.0%	59/59	100%
CCK/CCK-Br	6/6	100%	5/5	100%
CCK-Ar/CCK	6/10	60.0%	59/59	100%
CCK-Br/CCK	6/11	54.5%	5/26	19.2%

**Table 4:** Coexpression ratio of P_2_X_2_ and CCK receptor in the geniculate ganglion (GG) of mice.

**Marker**	**GG**	
CCK-Ar/P_2_X_2_	105/343	30.6%
P_2_X_2_/CCK-Ar	105/107	98.13%
CCK-Br/P_2_X_2_	46/210	21.9%
P_2_X_2_/CCK-Br	37/37	100%

### Reduction of gustatory nerve responses to bitter compounds in CCK receptor knockout mice

**Figure 5:**
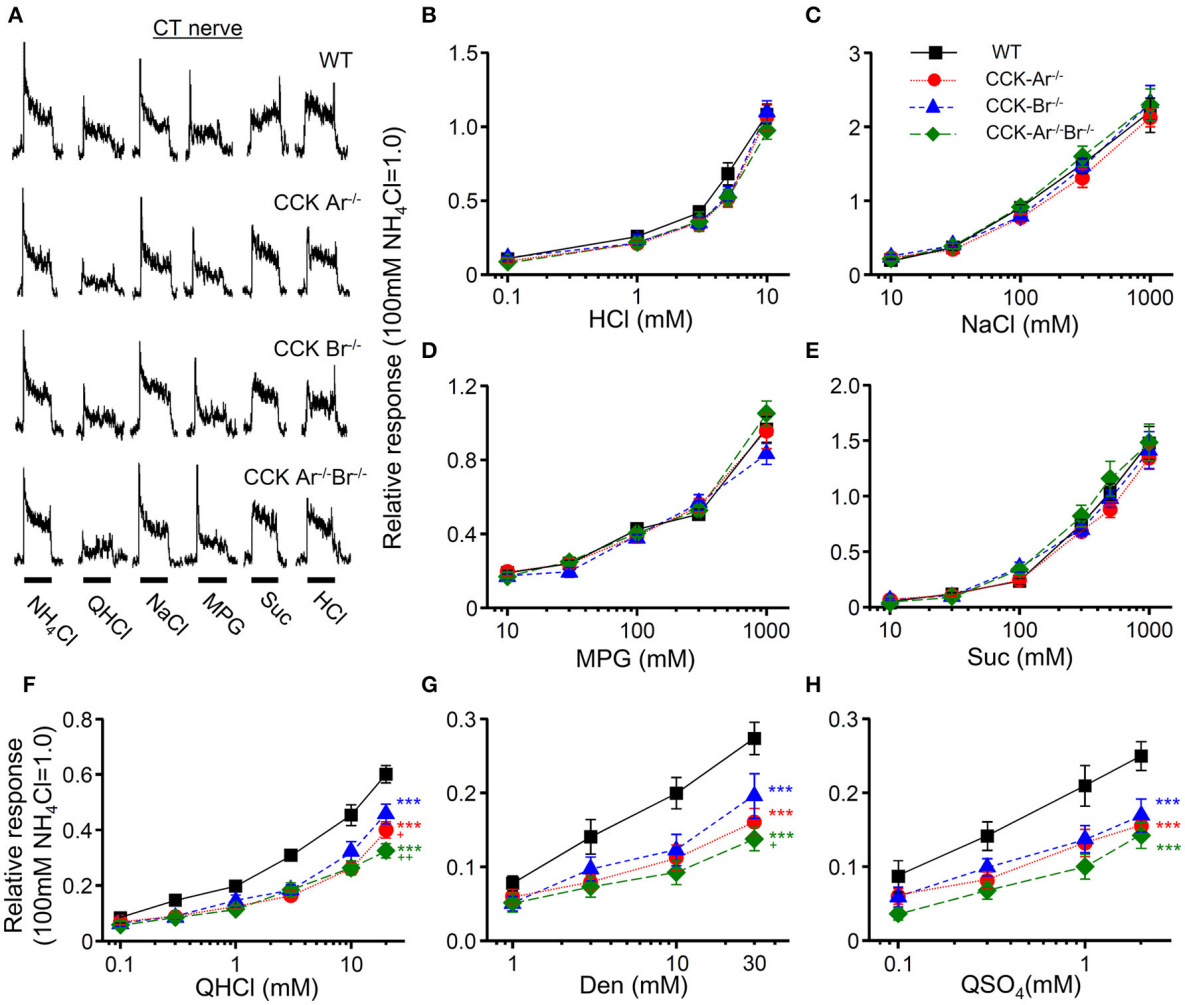
CT nerve responses to various tastants in WT, CCK-Ar^−/−^, CCK-Br^−/−^, and CCK-Ar^−/−^Br^−/−^ mice. **(A)** Typical examples of CT nerve responses to 100 mM NH_4_Cl (NH_4_Cl), 20 mM quinine-HCl (QHCl), 100 mM NaCl (NaCl), 100 mM monopottasium glutamate (MPG), 300 mM sucrose (Suc), and 10 mM HCl (HCl). Bars indicate taste stimulation (30 s). **(B–H)** Concentration response relationships of CT nerve responses for various tastants in WT, CCK-Ar^−/−^, CCK-Br^−/−^, and CCK-Ar^−/−^Br^−/−^ mice. Taste stimuli were 0.1–10 mM HCl **(B)**, 10–1,000 mM NaCl **(C)**, 10–1,000 mM MPG **(D)**, 10–1,000 mM Suc **(E)**, 0.1–20 mM QHCl **(F)**, 1–30 mM Den **(G)**, and 0.1–2 mM QSO_4_ **(H)**. CT nerve responses in WT (black, *n* = 7–13), CCK-Ar^−/−^ (red, *n* = 10–19), CCK-Br^−/−^ (blue, *n* = 7–14), and CCK-Ar^−/−^Br^−/−^ mice (green, *n* = 8–10) were normalized to the response to 100 mM NH_4_Cl. Values indicated are mean ± SEM. Statistical differences were analyzed by two way ANOVA (Supplemental Table 1) and *post-hoc* Tukey HSD test (^***^P < 0.001 vs. WT; ^+^*P* < 0.05, ^++^*P* < 0.01 vs. CCK-Br^−/−^).

To assess the contribution of CCK to taste signaling, CT and GL nerve responses to various tastants were recorded in WT, CCK-Ar^−/−^, CCK-Br^−/−^, and CCK-Ar^−/−^Br^−/−^ mice. All mice showed CT nerve responses to various tastants such as QHCl, NaCl, HCl, Suc, and MPG (Figure 5A), which were concentration dependent (Figures 5B-H). CT nerve responses to bitter tastants, such as QHCl (Figure 5F), Den (Figure 5G), and QSO_4_ (Figure 5H), were significantly different among WT, CCK-Ar^−/−^, CCK-Br^−/−^, and CCK-Ar^−/−^Br^−/−^ mice (two-way ANOVA, *P* < 0.001, effect of genotype, Supplemental Table 1). In contrast, significant differences were not observed in CT nerve responses to sour (HCl, Figure 5B), salty (NaCl, Figure 5C), umami (MPG, Figure 5D), or sweet (Suc, Figure 5E) tastants (two-way ANOVA, *P* > 0.05, effect of genotype, Supplemental Table 1). CT nerve responses to bitter compounds (QHCl, Den and QSO_4_) were significantly smaller in CCK-Ar^−/−^, CCK-Br^−/−^, and CCK-Ar^−/−^Br^−/−^ mice than in WT mice (*P* < 0.001, *post-hoc* Tukey HSD test, Figures 5F-H). CT nerve responses to QHCl were significantly smaller in CCK-Ar^−/−^ and CCK-Ar^−/−^Br^−/−^ mice than in CCK-Br^−/−^ mice (*P* < 0.05-0.01, *post-hoc* Tukey HSD test, Figure 5F). CT nerve responses to Den were significantly smaller in CCK-Ar^−/−^Br^−/−^ mice than in CCK-Br^−/−^ mice (*P* < 0.05, *post-hoc* Tukey HSD test, Figure 5G). Similar results were observed in GL nerve responses (Supplemental Figure 1, Supplemental Table 1).

**Figure 6:**
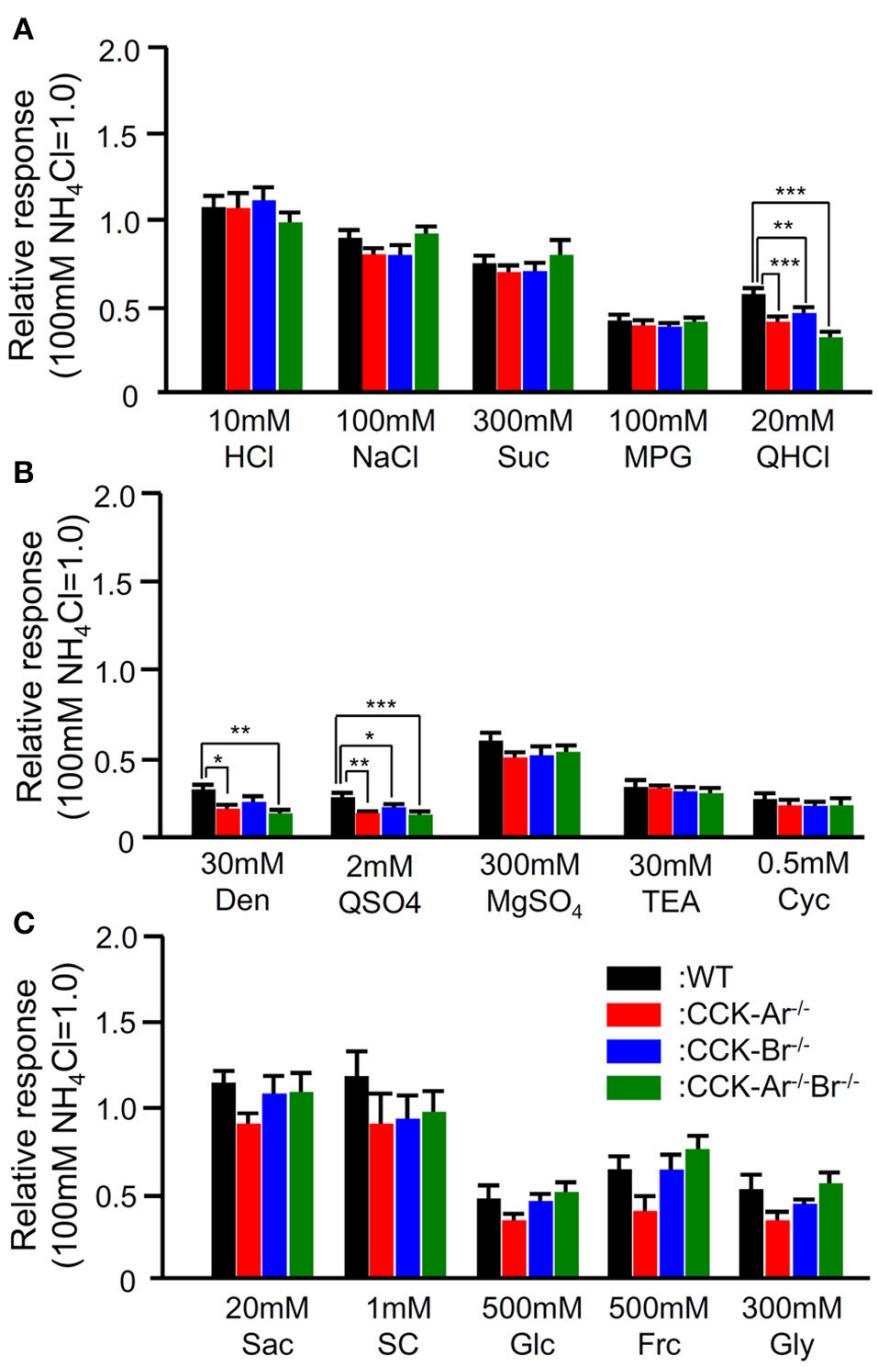
CT nerve responses to various tastants in WT, CCK-Ar^−/−^, CCK-Br^−/−^, and CCK-Ar^−/−^Br^−/−^ mice. **(A)** CT nerve responses to five basic tastants (10 mM HCl, 100 mM NaCl, 300 mM Suc, 100 mM MPG, and 20 mM QHCl) in WT, CCK-Ar^−/−^, CCK-Br^−/−^, and CCK-Ar^−/−^Br^−/−^ mice. **(B)** CT nerve responses to various bitter compounds (30 mM Den, 2 mM QSO_4_, 300 mM MgSO_4_, 30 mM TEA, and 0.5 mM Cyc) in WT, CCK-Ar^−/−^, CCK-Br^−/−^, and CCK-Ar^−/−^Br^−/−^ mice. **(C)** CT nerve responses to various sweet compounds (20 mM Sac, 1 mM SC, 500 mM Glc, 500 mM Frc, and 300 mM Gly) in WT, CCK-Ar^−/−^, CCK-Br^−/−^, and CCK-Ar^−/−^Br^−/−^ mice. CT nerve responses in WT (black, *n* = 5–11), CCK-Ar^−/−^ (red, *n* = 5–15), CCK-Br^−/−^ (blue, *n* = 5–13), and CCK-Ar^−/−^Br^−/−^ mice (green, *n* = 5–10) were normalized to the response to 100 mM NH_4_Cl. Values indicated are mean ± SEM. Statistical differences were analyzed by one way ANOVA (Supplemental Table 2) and *post-hoc* Tukey HSD test (^*^P < 0.05, ^**^P < 0.01, ^***^P < 0.001).

In addition, CT nerve responses to typical basic tastants (HCl, NaCl, Suc, MPG, QHCl; Figure 6A), various sweet compounds (Sac, SC, Glc, Frc, Gly; Figure 6B), and various bitter compounds (Den, QSO_4_, MgSO_4_, TEA, Cyc; Figure 6C) were compared among WT, CCK-Ar^−/−^, CCK-Br^−/−^, and CCK-Ar^−/−^Br^−/−^ mice. Among these tastants, some bitter compounds (QHCl, Den, QSO_4_) elicited smaller CT nerve responses in CCK-Ar^−/−^, CCK-Br^−/−^, and CCK-Ar^−/−^Br^−/−^ mice than in WT mice (one-way ANOVA, *P* < 0.01–0.001, Supplemental Table 2). CT responses to other tastants, including MgSO_4_, TEA, and Cyc were not significantly different among these mice (one-way ANOVA, *P* > 0.05, Supplemental Table 2). These results suggest that CCK is involved in normal gustatory responses to bitter compounds.

### Suppression of gustatory nerve responses to bitter stimuli by i.v. injection of CCK-Ar antagonist

**Figure 7:**
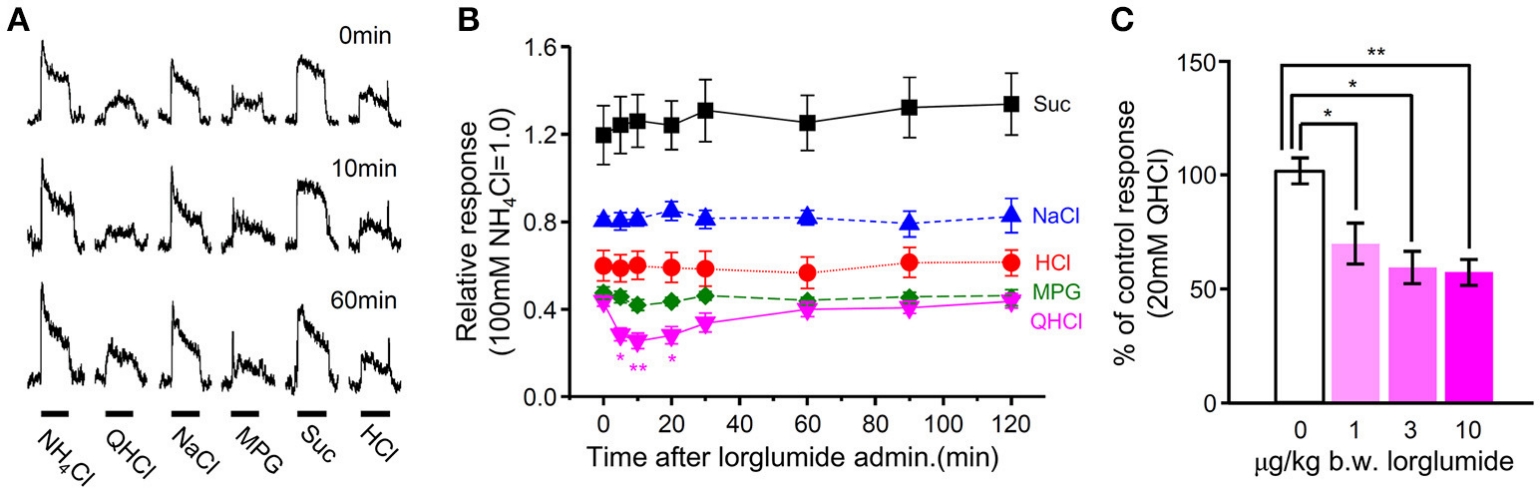
The effect of i.v. injection of lorglumide on CT nerve responses to taste stimuli. **(A)** Typical examples of CT nerve responses to 100 mM NH_4_Cl (NH_4_Cl), 20 mM quinine-HCl (QHCl), 100 mM NaCl (NaCl), 100 mM monopottasium glutamate (MPG), 300 mM sucrose (Suc), and 10 mM HCl (HCl) before (0 min), 10 min, and 60 min after administration of 10 μg/Kg b.w of lorglumide in a WT mouse. Bars indicate taste stimulation (30 s). **(B)** Time-dependent changes in CT nerve responses to 300 mM Suc (black), 100 mM NaCl (blue), 10 mM HCl (red), 100 mM MSG (green), and 20 mM QHCl (magenta) 0–120 min after administration of 10 μg/Kg b.w of lorglumide in WT mice (*n* = 6–7). **(C)** Dose-dependent effect of lorglumide administration on CT nerve responses to 20 mM QHCl in WT mice (*n* = 5–7). CT nerve responses to QHCl 5–20 min after administration of 0–10 μg/Kg b.w. of lorglumide were normalized to those before administration. Values indicated are mean ± SEM. Statistical differences were analyzed by one way ANOVA (Supplemental Table 3) and *post-hoc* Tukey HSD test (^*^P < 0.05, ^**^P < 0.01).

Next we determined whether the CCK receptor antagonist affected gustatory nerve responses to taste compounds. CT nerve responses to various tastants (300 mM Suc, 10 mM HCl, 100 mM NaCl, 100 mM MPG, and 20 mM QHCl) were recorded at 0–120 min after i.v. administration of CCK-Ar antagonist lorglumide. After i.v. administration of 10 μg/Kg b.w. lorglumide, CT nerve responses to QHCl were significantly reduced and then recovered to the control level (Figures 7A,B, Supplemental Table 3). CT nerve responses to Suc, HCl, NaCl, and MPG were not affected by administration of 10 μg/Kg b.w. lorglumide (Figures 7A,B, Supplemental Table 3). The effect of administration of lorglumide was dose-dependent (Figure 7C). These results were consistent with observations that CCK-Ar^−/−^ and CCK-Ar^−/−^Br^−/−^ mice showed reductions of neural responses to bitter compounds.

### Activation of gustatory nerves by i.v. injection of CCK

**Figure 8:**
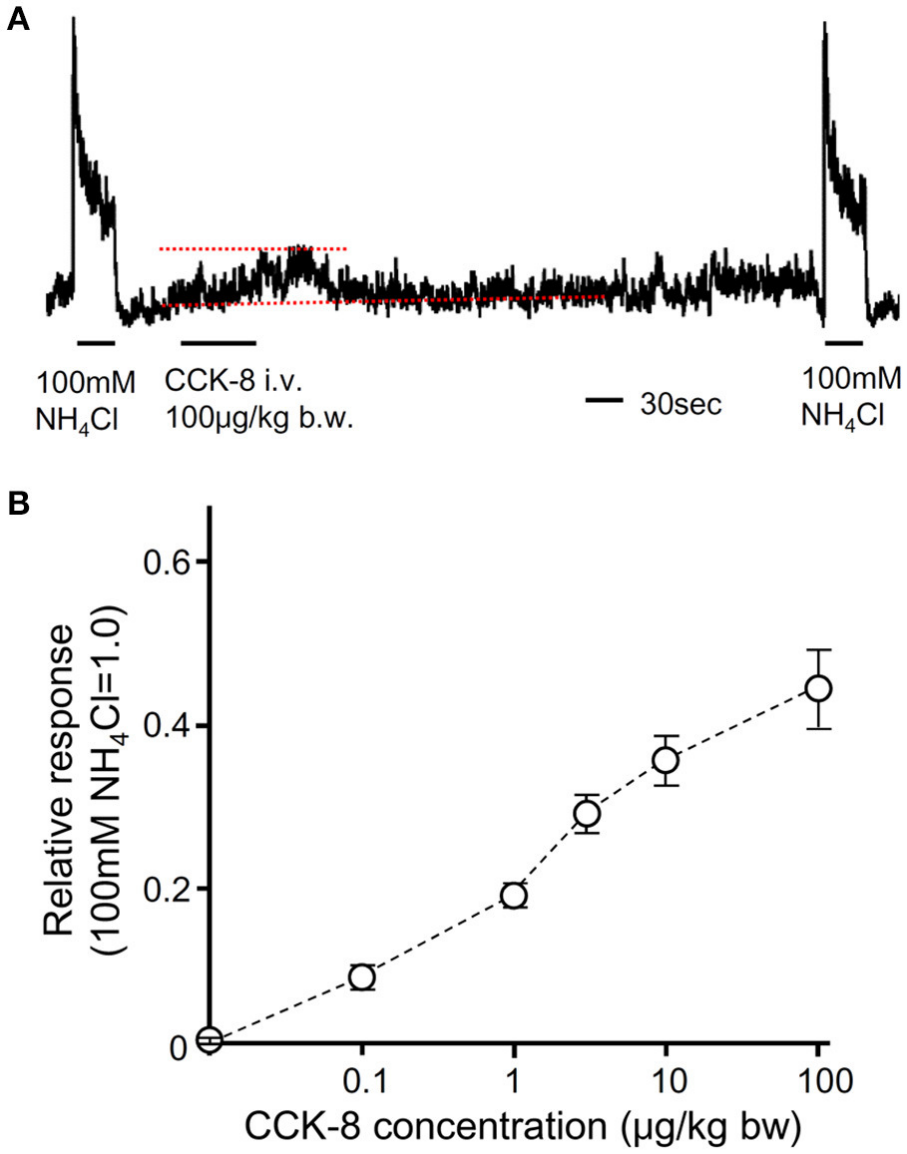
The effect of i.v. injection of CCK-8 on CT nerve activities. **(A)** A representative recording of CT nerve responses to 100 mM NH_4_Cl and i.v. injection of CCK-8. Dotted lines indicate baseline (lower) and peak amplitude (upper) of CT nerve activities after i.v. injection of CCK-8. 100 μg/Kg b.w. of CCK-8 was injected from femoral vein. **(B)** Concentration dependent CT nerve activities in response to i.v. injection of CCK-8 (0.1–100 μg/Kg b.w., *n* = 5). Values indicated are mean ± SEM.

To reveal the effect of CCK on gustatory nerve activities, we recorded CT nerve responses to i.v. injection of CCK-8. To analyze CT nerve activities elicited by i.v. injection of CCK-8, CT nerve responses to 100 mM NH_4_Cl were recorded before and after CCK-8 administration. Then, the peak amplitude of integrated whole nerve activities after i.v. injection of CCK-8 was normalized to responses to 100 mM NH_4_Cl (Figure 8A). After i.v. injection of CCK-8, integrated whole CT nerve activities were increased and then returned to the basal level at about 2 min after the end of injection (Figure 8A). CT nerve responses to i.v. injection of CCK-8 were dose-dependent (Figure 8B). These results suggest that CCK activates gustatory nerves.

### No significant effect of CCK on activities of bitter-sensitive taste cells

**Figure 9:**
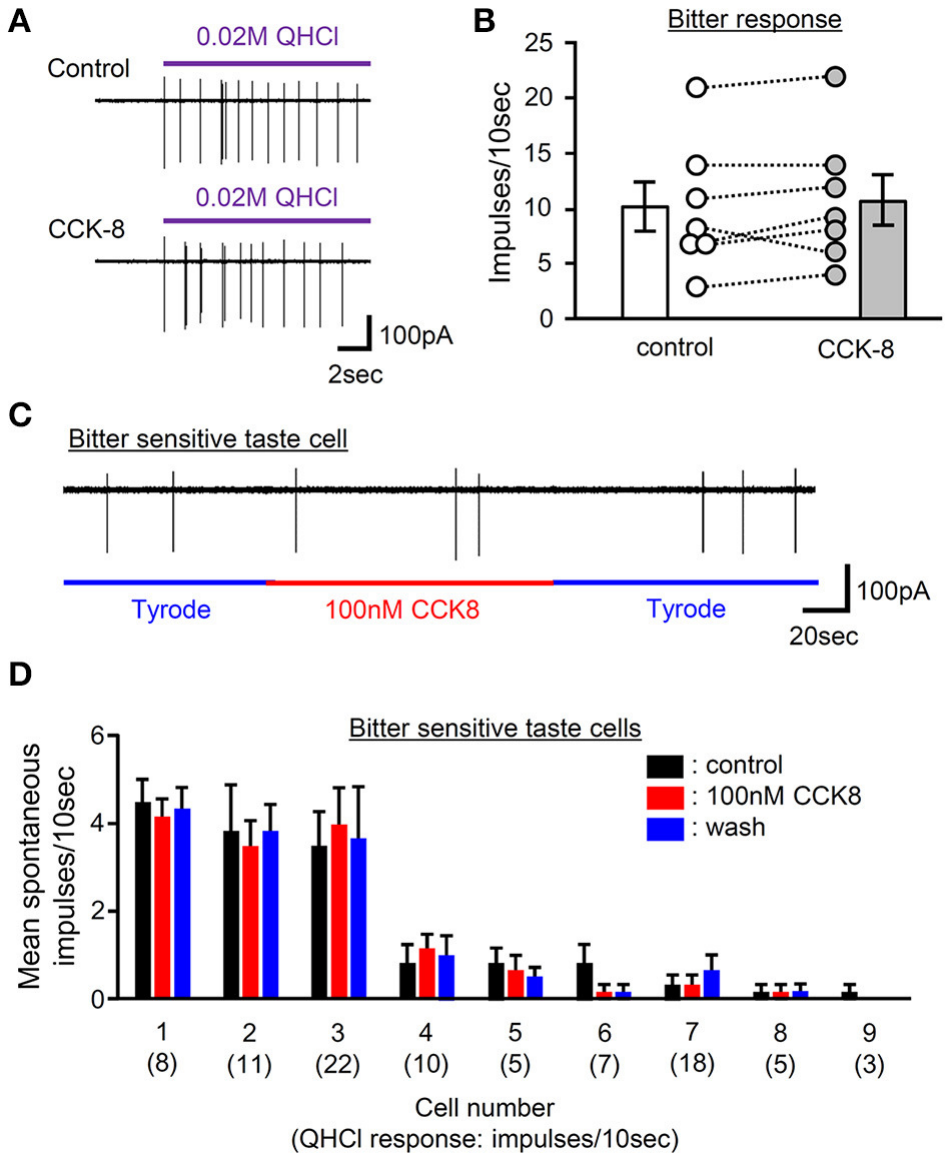
The effect of bath application of CCK-8 on activities of bitter-sensitive taste cells. **(A)** Sample recordings from a gustducin-GFP taste cell showing QHCl responses before (control) and 2 min after bath application of 100 nM CCK-8 (CCK-8). CCK-8 did not affect responses to QHCl in this cell. **(B)** Bitter taste responses of 7 individual gustducin-GFP taste cells before (control) and 2 min after bath application of 100 nM CCK-8 (CCK-8). Bars indicated are mean ± SEM (paired *t-*test, *P* > 0.1). **(C)** A sample recording from a bitter-sensitive gustducin-GFP taste cells showing the effect of bath application of CCK-8 on spontaneous activities. Bath application of 100 nM CCK-8 did not affect spontaneous activities of this cell. **(D)** Spontaneous firing activities of 9 individual bitter-sensitive taste cells before (black), during (red), and after (blue) bath application of 100 nM CCK-8. Values indicated are mean ± SEM. Statistical differences were analyzed by one way ANOVA [*F*_(2, 15)_ = 0 ~ 3.088, *P* > 0.05]. Magnitude of a response to 20 mM QHCl is shown in a parenthesis.

Our data indicate involvement of CCK in normal gustatory responses to bitter compounds. However, the site at which CCK receptor function is required remains unclear since CCK receptors are expressed in not only GG neurons but also taste cells (Figures 1, 3, 4). To determine the effect of CCK on taste cell activities, we recorded taste cell activities using an experimental setup which allows separate stimulation of the apical and basolateral faces of mouse fungiform taste cells. We focused on bitter-sensitive taste cells which could be identified by GFP expression in gustducin-GFP mice and responses to QHCl (Yoshida et al., 2009). As shown in Figures 9A,B, bath application of 100 nM CCK-8 did not affect bitter responses of taste cells. In addition, bath application of 100 nM CCK-8 did not induce any changes in spontaneous firing activities of bitter-sensitive taste cell (Figures 9C,D). These results indicate that CCK receptors expressed in bitter sensitive taste cells may not contribute to activities of these cells. Taken together, reduction of gustatory nerve responses to bitter compounds in CCK receptor knockout mice and CCK-induced activities of gustatory nerves may be mediated by CCK receptors expressed in GG neurons but not in bitter-sensitive taste cells.

## Discussion

Previous studies demonstrate that CCK and CCK-Ar are expressed in a subset of rat taste cells (Herness et al., 2002; Herness and Zhao, 2009). About 60% of CCK-positive taste cells express Gα-gustducin and about 15% of them express T1R3 (Herness et al., 2005). Our immunohistochemical data in mice are consistent with those in previous observations. In addition, we demonstrated that a subset of taste cells coexpressed CCK and CCK-Br (Figure 3) and CCK-positive taste cells expressed type II cell markers TRPM5 and PLCβ2 (Figure 2). Type II cells express receptors and transduction molecules for sweet, umami, and bitter tastes, and respond to sweet, umami and bitter compounds (Tomchik et al., 2007; Yoshida et al., 2009; Yoshida and Ninomiya, 2010). The majority (70–85%) of CCK-positive taste cells did not express the sweet/umami receptor component T1R3, indicating that these taste cells may be bitter-sensitive taste cells, while the rest (15–30%) may be sweet and/or umami-sensitive taste cells. Because there is no available antibody against mouse bitter receptors T2Rs to validate expression of T2Rs in taste tissue, the coexpression patterns of CCK and T2Rs are still unclear. Further investigations are required to reveal the molecular expression patterns in CCK- and CCK receptor-positive taste cells.

CCK-Ar and CCK-Br were also expressed in GG neurons (Figures 1, 4), implying that CCK released from taste cells activates gustatory nerve fibers expressing CCK receptors. Indeed, CT nerve activities were transiently increased after i.v. injection of CCK-8 (Figure 8). A previous study also reported that lingual artery injection of 10 μg CCK-8 elicited small increases in CT nerve activities in rats (Simon et al., 2003). We exclude the possibility that activation of taste cells expressing CCK receptors is involved in CCK-8-induced CT nerve activities, because administration of CCK-8 did not induce spike activities of bitter-sensitive taste cells (Figure 9). This result may not consistent with previous data showing CCK-induced Ca^2+^ responses and inhibition of K^+^ current in isolated rat taste cells (Herness et al., 2002; Lu et al., 2003). However, previous studies have not looked at spike activities of taste cells, which may be important for information transmission from taste cells to gustatory nerve fibers (Yoshida et al., 2006; Yoshida and Ninomiya, 2010). Taken together, CCK-8-induced CT nerve activities could be elicited by activation of CCK receptors expressed in gustatory nerve fibers.

CCK-Ar^−/−^ mice show a normal body weight, normal glucose tolerance (Takiguchi et al., 2002), and a high probability of gallstone formation, a concretion of bile components (Miyasaka et al., 2007). CCK-Br^−/−^ mice demonstrate greater daily energy intake and expenditure (Miyasaka et al., 2002a) as well as increases in anxiety-related behaviors (Miyasaka et al., 2002b). CCK-Ar^−/−^Br^−/−^ mice exhibit a lack of ghrelin secretion in response to fasting (Sakurai et al., 2006). However, taste sensitivities have not been investigated in these knockout mice. We demonstrated that bitter taste sensitivities were significantly smaller in CCK receptor knockout mice than in WT mice (Figures 5, 6, Supplemental Figure 1). In addition, i.v. injection of CCK-Ar antagonist lorgulmide selectively suppressed CT nerve responses to bitter stimuli (Figure 8). Thus, CCK may contribute to bitter taste sensitivity. It has been reported that CCK is released from the mouse enteroendocrine cell line STC-1 in response to bitter tastants (Chen et al., 2006; Jeon et al., 2008; Miyata et al., 2014). Although, we have no direct evidence for CCK release from taste cells because of insufficient sensitivity to measure CCK by commercially available ELISA kits, we speculate that CCK may be released from taste cells by bitter stimuli. Similar to the case of glucagon-like peptide-1 (GLP-1) in sweet taste signaling (Takai et al., 2015), released CCK may function as a specific neurotransmitter for bitter taste in peripheral taste signaling because some neurons in the GG expressed CCK receptors (Figure 4) and i.v. injection of CCK-8 induced transient CT nerve activities (Figure 7).

Regarding signal transmission in the peripheral taste system, the importance of ATP has been well established. Mice genetically lacking ATP receptors P_2_X_2_/P_2_X_3_ show markedly diminished nerve responses to all taste qualities (Finger et al., 2005). ATP is released from type II cells in response to sweet, bitter, or umami taste stimuli (Huang et al., 2007; Murata et al., 2010). Thus, ATP plays a crucial role in taste signal transmission. ATP and CCK may be co-released from taste cells in response to bitter compounds, and both of them may act on gustatory nerve fibers expressing CCK receptors because all CCK receptor-expressing neurons in the GG expressed P_2_X_2_. In this manner, CCK may function as an ancillary but functionally important neurotransmitter in cooperation with ATP, which might be required for maximal activation of bitter nerve fibers.

Taste bud cells not only express CCK, but also some bioactive peptides such as GLP-1, glucagon, neuropeptide Y (NPY), and vasoactive intestinal peptide (VIP). Among them, GLP-1 is involved in signal transmission of sweet taste from taste cells to gustatory nerve fibers (Takai et al., 2015). The function of CCK in bitter taste may correspond to that of GLP-1 in sweet taste. Glucagon is coexpressed with T1R3 and the glucagon receptor (Elson et al., 2010). Therefore, glucagon may act as a feedback signal in sweet-sensitive taste cells. NPY, VIP, and CCK are often coexpressed in taste cells (Zhao et al., 2005), suggesting that these peptides may be expressed in bitter-sensitive taste cells. Exogenous application of NPY activates K^+^ currents in isolated taste cells (Zhao et al., 2005) and the VIP receptor is expressed in type II cells (Shen et al., 2005). Therefore, NPY and VIP may function as autocrine or paracrine signals in taste buds. Although expression of receptors for glucagon, NPY, and VIP has not been elucidated in the GG, these peptides may be involved in signal transmission of particular taste qualities from taste cells to gustatory nerve fibers.

The effect of the lack of CCK receptors was greater on gustatory nerve responses to QHCl, QSO_4_, and Den than those to TEA, MgSO_4_, and Cyc (Figure 6). In rat CV taste buds, most bitter-sensitive taste cells responded to one of five tested bitter compounds (Caicedo and Roper, 2001). Expression analyses of mouse and human CV taste buds have demonstrated limited coexpression of T2Rs, suggesting heterogeneous populations of bitter sensitive taste cells (Matsunami et al., 2000; Behrens et al., 2007). Such evidence raises the possibility that bitter-sensitive taste cells responding to QHCl, QSO_4_, and Den and the gustatory nerve fiber innervating these taste cells may be different from those responding to TEA, MgSO_4_, and Cyc and nerve fibers innervating them, respectively, and expression patterns of CCK and CCK receptors may be different. The former taste cells and fibers express CCK and CCK receptors, whereas the later taste cells and fibers do not. Conversely, expression analysis of rat taste buds has demonstrated that a single taste receptor cell expresses large repertories of T2Rs (Adler et al., 2000). In this case, the amounts of released CCK in response to various bitter compounds from taste cells may be different.

Our data also demonstrate that some T1R3-positive taste cells express CCK. These cells may be sweet/umami sensitive taste cells. In the gut and STC-1 cells, CCK release induced by amino acids is mediated by T1R1/T1R3 (Daly et al., 2013). Therefore, it is possible that a subset of umami sensitive taste cells could secrete CCK in response to amino acids. Although, our data demonstrate that gustatory nerve responses to umami substance in CCK receptor knockout mice are not significantly different from WT mice, umami-induced CCK release from taste cells could activate gustatory nerve fibers. This possibility would be tested in the future studies.

In conclusion, we examined the function of CCK in the peripheral taste system. The coexpression patterns of CCK and taste cell markers suggest that the majority of CCK-expressing taste cells is likely to be bitter taste cells. Its receptors, CCK-Ar and CCK-Br, were expressed in both taste cells and gustatory neurons in the GG. Gustatory nerve responses to bitter compounds were smaller in CCK receptor knockout mice than in WT mice. In addition, intravenous injection of CCK-A receptor antagonist selectively suppressed gustatory nerve responses to bitter compounds. Intravenous injection of CCK agonist transiently increased gustatory nerve activities in a dose-dependent manner, whereas administration of CCK agonist did not affect activities of bitter sensitive taste cells. From these results, we speculate that CCK released from bitter sensitive taste cells activate gustatory neurons expressing CCK receptors. CCK may be a functionally important neurotransmitter or neuromodulator to activate bitter nerve fibers in peripheral taste tissues.

## Author contributions

RY and YN conceived and designed the experiments. RY, MS, KY, S.Takai, MI, and NS performed the experiments. RY, MS, KY, S.Takai, MI, NS, S.Takiguchi, SN, and YN analyzed the data. S.Takiguchi contributed reagents/materials/analysis tools. RY, MS, and YN wrote the paper. All authors approved the final version of the manuscript.

### Conflict of interest statement

The authors declare that the research was conducted in the absence of any commercial or financial relationships that could be construed as a potential conflict of interest.

## Supplementary material

Supplementary Figure 1 GL nerve responses to various tastants in WT, CCK-Ar^−/−^, CCK-Br^−/−^, and CCK-Ar^−/−^Br^−/−^ mice. **(A)** Typical examples of GL nerve responses to 100 mM NH_4_Cl (NH_4_Cl), 20 mM quinine-HCl (QHCl), 100 mM NaCl (NaCl), 100 mM monopottasium glutamate (MPG), 300 mM sucrose (Suc), and 10 mM HCl (HCl) in WT, CCK-Ar^−/−^, CCK-Br^−/−^, and CCK-Ar^−/−^Br^−/−^ mice. Bars indicate taste stimulation (60 s). **(B–H)** Concentration response relationships of GL nerve responses for various tastants in WT, CCK-Ar^−/−^, CCK-Br^−/−^, and CCK-Ar^−/−^Br^−/−^ mice. Taste stimuli were 0.1–10 mM HCl **(B)**, 10–10,00 mM NaCl **(C)**, 10–1,000 mM MPG **(D)**, 30–1,000 mM Suc **(E)**, 0.1–10 mM QHCl **(F)**, 1–20 mM Den **(G)**, and 0.1–2 mM QSO_4_ **(H)**. GL nerve responses in WT (black, *n* = 8–18), CCK-Ar^−/−^ (red, *n* = 7–14), CCK-Br^−/−^ (blue, *n* = 6–16), and CCK-Ar^−/−^Br^−/−^ mice (green, *n* = 7–13) were normalized to the response to 100 mM NH_4_Cl. Values indicated are mean ± SEM. Statistical differences were analyzed by two way ANOVA (Supplemental Table 1) and *post-hoc* Tukey HSD test (^*^*P* < 0.05, ^**^*P* < 0.01, ^***^*P* < 0.001 vs. WT; ^+^*P* < 0.05, ^++^*P* < 0.01 vs. CCK-Br^−/−^).

## Abbreviations

CCK: cholecystokinin
CT: chorda tympani
CV: circumvallate papillae
Cyc: cyclohexamide
DIG: digoxigenin
DW: distilled water
Frc: fructose
FP: fungiform papillae
GFP: green fluorescent protein
GG: geniculate ganglion
GL: glossopharyngeal
Glc: glucose
GLP-1: glucagon-like peptide-1
Gly: glycine
HSD: honestly significant difference
MPG: monopotassium glutamate
NPY: neuropeptide Y
PFA: paraformaldehyde
PLCβ2: phospholipase Cβ2
QHCl: quinine-HCl
QSO_4_: quinine-SO_4_
Sac: saccharin-Na
SC: SC45647
SSC: standard saline citrate
Suc: sucrose
T1R1 (or T1R3): taste receptor type 1 member 1 (or 3)
T2R: taste receptor type 2
TEA: tetraethylammonium
TRPM5: transient receptor potential channel M5
TBS: Tris-buffered saline
VIP: vasoactive intestinal peptide
WT: wild-type.
