# Supplementary figures and images for: The Role of Cholecystokinin in Peripheral Taste Signaling in Mice
Source: Front Physiol. 2017 Oct 31;8:866. doi: 10.3389/fphys.2017.00866 (PMC5671461; doi:10.3389/fphys.2017.00866)

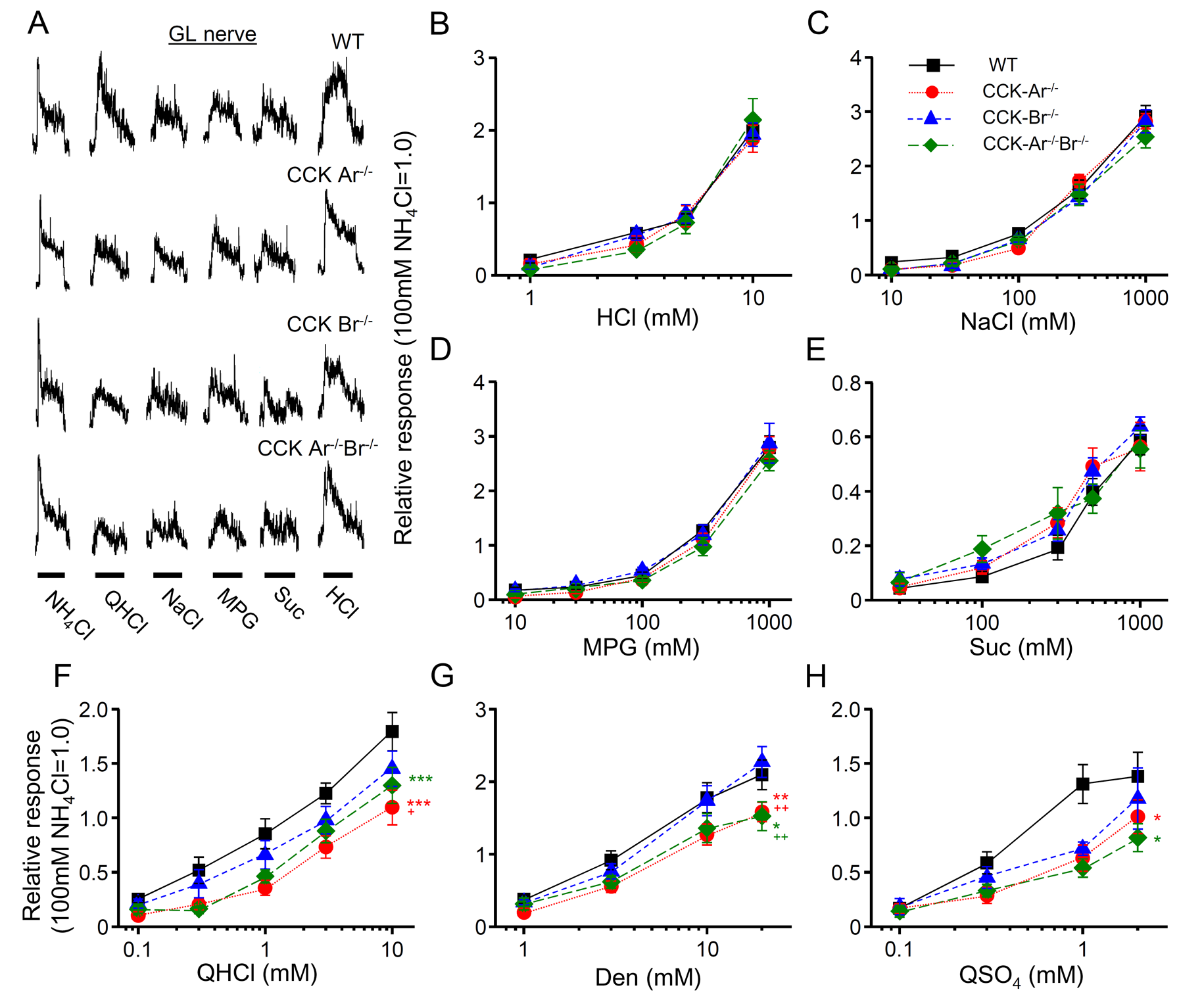

Supplement: Supplementary Figure 1 — GL nerve responses to various tastants in WT, CCK-Ar−/−, CCK-Br−/−, and CCK-Ar−/−Br−/− mice. (A) Typical examples of GL nerve responses to 100 mM NH4Cl (NH4Cl), 20 mM quinine-HCl (QHCl), 100 mM NaCl (NaCl), 100 mM monopottasium glutamate (MPG), 300 mM sucrose (Suc), and 10 mM HCl (HCl) in WT, CCK-Ar−/−, CCK-Br−/−, and CCK-Ar−/−Br−/− mice. Bars indicate taste stimulation (60 s). (B–H) Concentration response relationships of GL nerve responses for various tastants in WT, CCK-Ar−/−, CCK-Br−/−, and CCK-Ar−/−Br−/− mice. Taste stimuli were 0.1–10 mM HCl (B), 10–10,00 mM NaCl (C), 10–1,000 mM MPG (D), 30–1,000 mM Suc (E), 0.1–10 mM QHCl (F), 1–20 mM Den (G), and 0.1–2 mM QSO4 (H). GL nerve responses in WT (black, n = 8–18), CCK-Ar−/− (red, n = 7–14), CCK-Br−/− (blue, n = 6–16), and CCK-Ar−/−Br−/− mice (green, n = 7–13) were normalized to the response to 100 mM NH4Cl. Values indicated are mean ± SEM. Statistical differences were analyzed by two way ANOVA (Supplemental Table 1) and post-hoc Tukey HSD test (*P < 0.05, **P < 0.01, ***P < 0.001 vs. WT; +P < 0.05, ++P < 0.01 vs. CCK-Br−/−). [file Image1.TIF]
