# Supplementary material for: The Role of Cholecystokinin in Peripheral Taste Signaling in Mice
Source: Front Physiol. 2017 Oct 31;8:866. doi: 10.3389/fphys.2017.00866 (PMC5671461; doi:10.3389/fphys.2017.00866)
Supplement: Supplementary file 2 [file Table1.pdf]

Supplemental Table 1

Two-way ANOVA results for CT and GL nerve responses to various tastents (WT, CCK-Ar$^{-/-}$, CCK-Br$^{-/-}$, vs CCK-Ar$^{-/-}$Br$^{-/-}$)

	Effect of genotype			
	CT nerve		GL nerve	
tastants	DF	F	DF	F
QHCl	3,258	38.8***	3.192	10.5***
Den	3,151	18.1***	3,135	7.4***
QSO_4	3,146	19.9***	3,119	6.6***
HCl	3,260	2.3	3,142	0.4
NaCl	3,203	1.6	3,191	1.7
MPG	3,184	1.2	3,162	2.3
Suc	3,229	1.8	3,154	1.0

Table based on data shown in Fig. 5 and supplemental Fig. 1. DF: degree of freedom. F: F values. ***: P<0.001, ANOVA.
