# Supplementary material for: The Role of Cholecystokinin in Peripheral Taste Signaling in Mice
Source: Front Physiol. 2017 Oct 31;8:866. doi: 10.3389/fphys.2017.00866 (PMC5671461; doi:10.3389/fphys.2017.00866)
Supplement: Supplementary file 3 [file Table2.pdf]

Supplemental Table 2

One-way ANOVA results for CT nerve responses to various tastents (WT, CCK-Ar$^{-/-}$, CCK-Br$^{-/-}$, vs CCK-Ar$^{-/-}$Br$^{-/-}$)

	Effect of genotype	
tastants	DF	F
QHCl	3,46	12.2***
Den	3,35	6.6**
QSO_4	3,38	8.5***
HCl	3,35	0.6
NaCl	3,43	2.0
MPG	3,39	0.5
Suc	3,41	1.1
$MgSO_4$	3,39	1.3
TEA	3,42	2.4
Cyc	3,31	0.4
Sac	3,31	1.3
SC	3,22	0.7
Glc	3,21	2.0
Frc	3,19	2.5
Gly	3,20	2.9

Table based on data shown in Fig. 6. DF: degree of freedom. F: F values. **: P<0.01, ***: P<0.001, ANOVA.
