# Supplementary material for: The Role of Cholecystokinin in Peripheral Taste Signaling in Mice
Source: Front Physiol. 2017 Oct 31;8:866. doi: 10.3389/fphys.2017.00866 (PMC5671461; doi:10.3389/fphys.2017.00866)
Supplement: Supplementary file 4 [file Table3.pdf]

Supplemental Table 3

One-way ANOVA results for time dependent changes in CT nerve responses after lorglumide administration

	Effect of lorglumide	
tastants	DF	F
QHCl	7,47	4.9***
HCl	7,40	0.1
NaCl	7,40	0.1
MPG	7,47	0.5
Suc	7,46	0.1

The effects of lorglumide were analyzed by owo-way ANOVA. Table based on data shown in Fig. 7. DF: degree of freedom. F: F values. ***: P<0.001, ANOVA.
